# Improved Operation of Chloralkaline Reversible Cells with Mixed Metal Oxide Electrodes Made Using Microwaves

**DOI:** 10.3390/nano14080693

**Published:** 2024-04-17

**Authors:** Jamylle Y. C. Ribeiro, Gessica O. S. Santos, Aline R. Dória, Iñaki Requena, Marcos R. V. Lanza, Giancarlo R. Salazar-Banda, Katlin I. B. Eguiluz, Justo Lobato, Manuel A. Rodrigo

**Affiliations:** 1 Electrochemistry and Nanotechnology Laboratory, Institute of Technology and Research (ITP), Aracaju 49032-490, SE, Brazil; jamyllerib@gmail.com (J.Y.C.R.); alinerdoria@gmail.com (A.R.D.); gianrsb@gmail.com (G.R.S.-B.); katlinbarrios@gmail.com (K.I.B.E.); 2 Graduate Program in Processes Engineering (PEP), Tiradentes University, Aracaju 49032-490, SE, Brazil; 3 Chemical Engineering Department, Faculty of Chemical Sciences and Technologies, Universidad Castilla-La Mancha, 13004 Ciudad Real, Spain; gessicasantiago@usp.br (G.O.S.S.); inaki.requena@uclm.es (I.R.); 4 São Carlos Institute of Chemistry, University of São Paulo, São Carlos 13566-590, SP, Brazil; marcoslanza@usp.br

**Keywords:** reversible electrochemical cells, mixed metal oxide, microwave, platinum, liquid–liquid fuel cell

## Abstract

This study focuses on the synthesis of mixed metal oxide anodes (MMOs) with the composition Ti/RuO_2_Sb_2_O_4_Pt*_x_* (where *x* = 0, 5, 10 mol) using hybrid microwave irradiation heating. The synthesized electrodes were characterized using scanning electron microscopy, X-ray energy-dispersive analysis, X-ray diffraction, cyclic voltammetry, and electrochemical impedance spectroscopy. These electrodes were then evaluated in both bulk electrolytic and fuel cell tests within a reversible chloralkaline electrochemical cell. The configurations using the electrodes Ti/(RuO_2_)_0.7_-(Sb_2_O_4_)_0.3_ and Ti/(RuO_2_)_66.5_-(Sb2O_4_)_28.5_-Pt_5_ presented lower onset potential for oxygen and chlorine evolution reactions and reduced resistance to charge transfer compared to the Ti/(RuO_2_)_63_-(Sb_2_O_4_)_27_-Pt_10_ variant. These electrodes demonstrated notable performance in reversible electrochemical cells, achieving Coulombic efficiencies of up to 60% when operating in the electrolytic mode at current densities of 150 mA cm^−2^. They also reached maximum power densities of 1.2 mW cm^−2^ in the fuel cell. In both scenarios, the presence of platinum in the MMO coating positively influenced the process. Furthermore, a significant challenge encountered was crossover through the membranes, primarily associated with gaseous Cl_2_. This study advances our understanding of reversible electrochemical cells and presents possibilities for further exploration and refinement. It demonstrated that the synergy of innovative electrode synthesis strategies and electrochemical engineering can lead to promising and sustainable technologies for energy conversion.

## 1. Introduction

Mixed metal oxide (MMO) electrodes have emerged as a cost-effective alternative to mitigate the costs associated with the utilization of noble metals. These electrodes offer the advantage of being more stable and durable than their single-component metal oxide equivalents [1,2]. The versatility of MMO electrodes, characterized by their adjustable physicochemical properties acquired through varying synthesis methods and morphologies, has contributed to their applications in various industries, including oxygen production, chlor-alkali generation [3], cathodic protection [4], and organic compound degradation [5,6].

In the fabrication of MMO anodes, a layer of desired mixed metallic oxides is deposited onto a substrate, often employing cost-effective titanium, and subsequently subjected to calcination, crucial for oxide formation [7,8]. Notably, the temperature of calcination is dependent upon the choice of metallic precursors and solvents. Although traditional furnaces are widely used in the calcination process, recent investigations have highlighted the efficacy of alternative heating methods [9,10], such as hybrid microwave heating, in enhancing anode stability and electrocatalytic performance. This innovation reduces the processing time and energy involved in the manufacturing of MMOs, leading to a more cost-effective and sustainable process. Furthermore, hybrid microwave irradiation accelerates diffusion mechanisms and improves mechanical properties, promoting elevated surface areas and catalytic activities [10].

The electrode preparation method employed in this study used microwave (MW) heating, which presents distinct advantages over traditional heating approaches. These include uniform thermal distribution throughout the sample, a factor critical to the homogeneity and performance consistency of electrodes. Additionally, MW heating is characterized by its energy efficiency, leading to reduced electricity consumption during the synthesis process. These attributes not only enhance the overall efficiency of electrode fabrication but also contribute to the environmental sustainability and cost-effectiveness of the method, aligning with broader objectives of resource conservation and economic viability in materials manufacturing [9]. In microwave hybrid heating, the system employs a susceptor material with a high microwave absorption capacity embedded within a matrix characterized by low dielectric loss. This configuration allows the microwaves to pass through the matrix with minimal absorption, focusing the energy on the susceptor. The susceptor, upon absorbing the microwave energy, rapidly heats up and subsequently transfers this heat to the sample surrounding material.

Moreover, the use of ionic liquids in the synthesis process, where specific ionic liquids act as solvents, confers several advantages. This approach enables the formation of oxide at relatively low temperatures, ensuring simplicity in synthesis, reproducibility, and controlled effects, such as stoichiometry and homogeneity [11]. Santos et al. [10] have investigated the application of thermal decomposition using ionic liquids, particularly the 2-hydroxyethyl ammonium acetate ionic liquid, for the preparation of the precursor solution. Their study revealed a remarkable ten-fold increase in the viscosity of the precursor solution (greater than 2.24 Pa s) compared to the other ionic liquid studied (methylimidazolium hydrogen sulfate). This increase in viscosity may enhance charge transfer rates while promoting a more porous morphology and facilitating larger surface areas for oxidation reactions.

On the other hand, the chlor-alkali method is an electrolytic process that produces mainly chlorine in the anode and caustic soda as well as hydrogen in the cathode while consuming brine and electricity [12]. RuO_2_-based MMO electrodes are promising for chlorine production due to their exceptional properties, including high conductivity, high thermal and chemical stability, suitability for reversible redox reactions, and comparative affordability compared to materials such as platinum [13,14,15]. Nevertheless, their susceptibility to corrosion under rigorous chlorine and oxygen release conditions during chloralkaline processes poses a challenge [10,16]. In order to address this issue, RuO_2_ is incorporated into composite oxides to improve corrosion resistance and coating stability [17]. In particular, antimony emerges as a favorable candidate due to its high electrical conductivity, thermodynamic stability at low pH, and natural abundance [18,19,20].

Commercially, ruthenium oxide-based MMOs have found utility as electrocatalysts for the chlorine evolution reaction (CER) within the chlorine-alkali industry (Ti/RuO_2_-TiO_2_) [21,22]. Recent research by Carvela et al. [23,24] has demonstrated the feasibility of ternary MMOs such as Ti/RuO_2_-TiO_2_-Pt; however, it is imperative to minimize platinum content due to its elevated market value. Therefore, the exploration of alternative metals to improve catalytic efficiency and reduce material costs is crucial [25].

In this study, we propose to evaluate the operational efficiency and stability of a chlor-alkali-reversible electrochemical cell employing three different ternary mixed metal oxide electrodes containing ruthenium, antimony, and low amounts of platinum. The synthesis of these electrodes used 2-hydroxyethylammonium acetate (2HEAA) as the solvent within the ionic liquid method for dissolving metallic precursors. The selection of 2HEAA as an IL is pivotal, as it significantly enhances the electrochemical attributes of the electrodes produced. This enhancement includes an increase in voltammetric charge capacity, improved resistance against charge transfer, extension of the electrode’s operational service life, decrease in the time required for synthesis, and increased stability of the electrodes. The synthesis involves hybrid heating via microwave irradiation (MW-MMO electrodes). Consequently, this work develops three anode compositions: (Ti/(RuO_2_)_70_-(Sb_2_O_4_)_30_, Ti/(RuO_2_)_66.5_-(Sb_2_O_4_)_28.5_-Pt_5_ and Ti/(RuO_2_)_63_-(Sb_2_O_4_)_27_-Pt_10_. We hypothesize that employing the proposed methodology to prepare these newly developed ternary mixed metal oxide electrodes will enhance efficiency and stability in operating a chlor-alkali reversible electrochemical cell.

## 2. Materials and Methods

### 2.1. Chemicals

All chemical reagents used in this study were of analytical grade and were used without further purification. Ruthenium (III) chloride hydrate (RuCl_3_xH_2_O–99.5%), antimony (III) chloride (SbCl_3_–99.0%), hexachloroplatinic acid (H_2_PtCl_6_·6H_2_O–37.5%), oxalic acid (C_2_H_2_O_4_–99.5%), and isopropanol (C_3_H_8_O–99.8%) were purchased from Sigma-Aldrich^®^. (Burlington, MA, USA). Hydrochloric acid (HCl–38.0%) was acquired from Neon^®^ (Suzana, São Paulo, Brazil), sulfuric acid (H_2_SO_4_–97%) from Scharlab^®^ (Sentmenat, Spain), and sodium chloride (NaCl–99.5%) from Panreac^®^ (ITW, Chicago, IL, USA). All solutions were prepared using ultrapure water (18.2 MΩcm, 25 °C) from a Gehaka Master All 2000 System (São Paulo, Brazil).

### 2.2. Preparation of MMO Anodes

**Figure 1:**
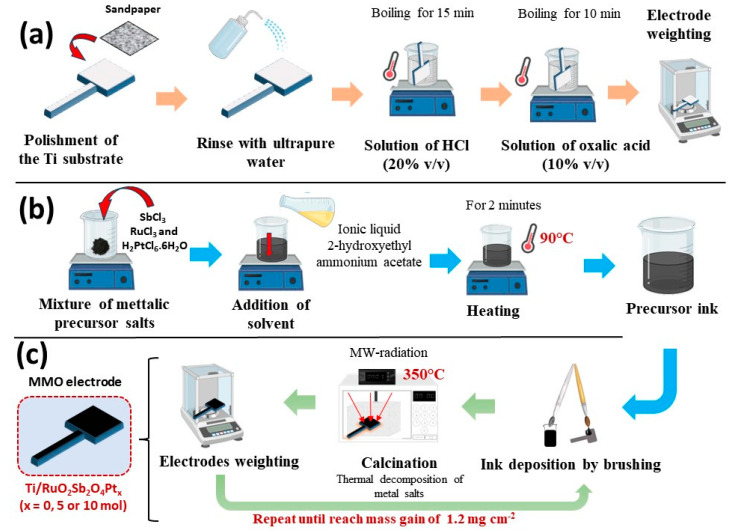
Schematic representation of the electrode preparation procedure using microwave hybrid heating and ionic liquid methods. (**a**) pretreatment of titanium plates, (**b**) preparation of the precursor solution, and (**c**) subsequent calcination processes.

Physical and chemical pretreatment of titanium plates was carried out as described by Santos et al. [10]. Subsequent to pretreatment, titanium substrates were coated with precursor solutions previously formulated to achieve catalytic coatings with the following compositions and proportions: (RuO_2_)_70_-(Sb_2_O_4_)_30_, Ti/(RuO_2_)_66.5_-(Sb_2_O_4_)_28.5_-Pt_5_ and Ti/(RuO_2_)_63_-(Sb_2_O_4_)_27_-Pt_10_. These precursor solutions were prepared using the liquid ionic (IL) method, as described by Santos et al. [10]. The 2HEAA LI employed as the solvent of the metallic salts exhibited an acidic pH of approximately 1. The metallic precursors employed included RuCl_3_, SbCl_3_, and H_2_PtCl_6_·6H_2_O. Subsequently, the prepared precursor solution was deposited onto pretreated titanium plates for subsequent calcination. The brushing and calcination processes were iterated between 8 and 11 times until the electrode reached the desired oxide mass gain of 1.2 mg cm^−2^ [11]. The final calcination temperature for the electrodes was maintained at 350 °C. Figure 1 illustrates the procedural schematic for electrode preparation, which covers the following steps: (a) pretreatment of titanium plates, (b) preparation of the precursor solution, and (c) subsequent calcination processes.

### 2.3. Electrochemical Setup

Figure S1 shows the cell configuration used throughout this study, operative in both electrolysis and fuel cell modes. In the fuel cell configuration, the polarity was reversed. The experimental setup involves two compartments separated by a Nafion^®^ 117 proton exchange membrane (The Chemours Company™, Wilmington, DE, USA). The anode consisted of a 1 cm^2^ Ti/(RuO_2_)*_x_*-(Sb_2_O_4_)*_y_*-Pt*_z_* (*x*:*y*:*z* = 70:30:0, 66.5:28.5:5 and 63:27:10), while the cathode was a lead oxide-coated Ti electrode (1 cm^2^). In electrolysis mode, both cell compartments were supplied with a concentrated NaCl solution (2.0 mol L^−1^), continuously circulated using a peristaltic pump with two channels for each cell compartment. Chlorine gas (Cl_2_) was accumulated in the anode gasometer, while hydrogen gas (H_2_) and sodium hydroxide (NaOH) were collected in the cathode gasometer. Gas accumulation was determined using a millimeter ruler.

In fuel cell mode, polarity reversal prompted hydrogen from the cathode gasometer to be injected into the cell, yielding electricity. The voltage values were collected using a multimeter.

### 2.4. Chemical Analysis

The samples were periodically collected from the anode and cathode compartments at 20 min intervals to quantify the chlorine reacted. The pH and conductivity measurements were performed using a regularly calibrated GLP22 Crison pH meter and a GLP31 Crison conductivity meter. For absorbance measurements, 3 mL of the liquid phase sample, supplemented with 50 µL of concentrated NaOH (3 mol L^−1^) and 5 mL of the withdrawn gas-phase sample mixed with 5 mL of concentrated NaOH (3 mol L^−1^), was employed. Absorbance measurements were conducted using an Agilent Cary Series 300 UV-Vis Spectrophotometer at a wavelength of 293 nm. In-depth information on chemical characterization can be found elsewhere [16].

### 2.5. Physical Characterization of Electrodes

The surface morphology of the anodes was visualized using a field emission scanning electron microscope (FE-SEM; Zeiss Gemini SEM 500, Oberkochen, Germany). The elemental chemical composition of the anodes was determined through the use of energy-dispersive X-ray spectroscopy (EDS) integrated with scanning electron microscopy (SEM) equipment. The crystallographic phases present within the MMO anodes were identified via X-ray diffraction (XRD) on a Philips PW-1700 diffractometer using CuKα radiation (λ = 1.5418 Å) at a scanning rate of 2° min^−1^ from 20° to 80°. XRD patterns were analyzed using the X’pert High Score Plus software (version 3.7), and the data obtained were cross-referenced with the Joint Committee on Powder Diffraction Standards (JCPDS) database.

### 2.6. Electrochemical Characterization of Electrodes

Electrochemical measurements were performed within a one-compartment Pyrex^®^ borosilicate glass cell using a conventional three-electrode configuration. The setup included a working electrode consisting of Ti/(RuO_2_)_70_-(Sb_2_O_4_)_30_, Ti/(RuO_2_)_66.5_-(Sb_2_O_4_)_28.5_-Pt_5_, and Ti/(RuO_2_)_63_-(Sb_2_O_4_)_27_-Pt_10_ (2 cm^2^), a graphite counter electrode, and an Ag/AgCl reference electrode. Electrochemical experiments were carried out using a potentiostat/galvanostat (AUTOLAB-PGSTAT302N). Cyclic voltammograms were acquired in a potential range of 0.0 to 1.4 V (vs. Ag/AgCl) at a scan rate of 50 mV s^−1^.

Linear sweep voltammetry (LSV) experiments were performed from 0.0 to 1.5 V (vs. Ag/AgCl) at a scan rate of 20 mV s^−1^ to identify changes in electrode catalytic performance in the onset potentials of oxygen (OER) and chlorine (CER) evolution reactions before and after chronoamperometric tests. These tests were performed in solutions of 0.5 mol L^−1^ H_2_SO_4_ and 2.0 mol L^−1^ NaCl.

Chronoamperometric measurements were conducted with a fixed potential of 1.4 V (vs. Ag/AgCl), over 21.600 s, in 2.0 mol L^−1^ NaCl, which approximates the operating conditions of the fuel cell. Electrochemical impedance spectroscopy (EIS) measurements were also performed to assess the ohmic and charge transfer resistances of the prepared MMO electrodes. EIS measurements were obtained by applying a predefined potential to OER and CER derived from LSV analyses, with a sinusoidal interference signal amplitude of 5 mV and a frequency range of 10,000 to 0.1 Hz, employing a logarithmic distribution of 10 frequencies per decade for electrodes immersed in both solutions of 0.5 mol L^−1^ H_2_SO_4_ and 2.0 mol L^−1^ NaCl.

## 3. Results and Discussion

### 3.1. Characterization of Electrodes

**Figure 2:**
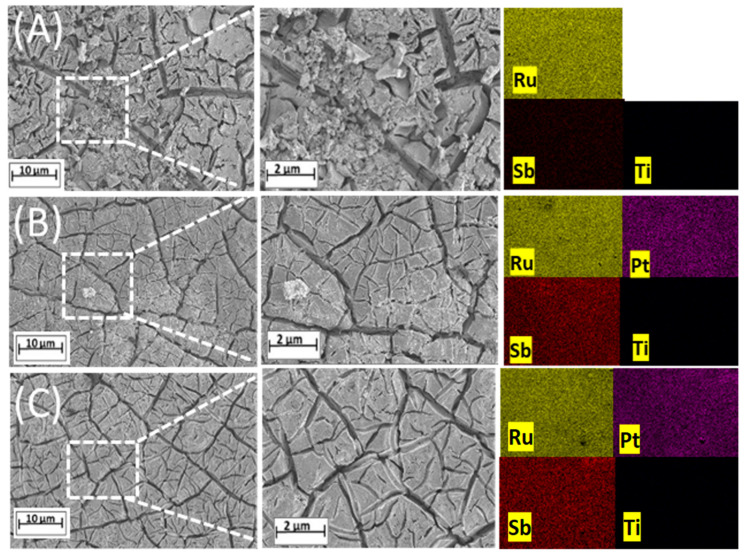
Micrographs obtained by MEV of two electrodes prepared using microwave hybrid heating: (**A**) Ti/(RuO_2_)_70_-(Sb_2_O_4_)_30_, (**B**) Ti/(RuO_2_)_66.5_-(Sb_2_O_4_)_28.5_-Pt_5_, and (**C**) Ti/(RuO_2_)_63_-(Sb_2_O_4_)_27_-Pt_10_.

The three MMO anodes studied here were synthesized using a unique technique that involved hybrid heating by microwave irradiation [25]. An exhaustive characterization of these electrodes was carried out before their performance in the electrolyzer and fuel cell setup was verified. Figure 2 shows the SEM images of the electrodes; regardless of the platinum content, the microwave technique produces coatings with a characteristic morphology of ‘cracked mud’ [26]. According to studies in the literature [10,26], the approach involving hybrid heating is efficient in promoting superior properties such as high surface area, elevated catalytic activity, and increased physical and mechanical stability, in addition to extended operational life compared to electrodes made using conventional fabrication methods.

The EDX results confirmed the presence of Ru, Sb, and Pt elements within the electrodes, along with their respective ratios. Inductively coupled plasma atomic emission spectroscopy (ICP-AES) is a spectroscopic technique used to quantify percentages of metal mass [21]. The elemental compositions were further examined using ICP-AES.

Table 1 shows the nominal and real composition values, revealing a notable correlation between the nominal compositions and those detected via EDX and ICP-AES analyses. The elemental mapping of each investigated anode demonstrated the uniform distribution of metals across the coatings. The different colors shown in the images correspond to different elemental compositions, while the alterations in brightness correlate with varying element concentrations.

X-ray diffractograms are shown in Figure S2. XRD peaks were indexed with the Joint Committee on Powder Diffraction Standards (JCPDS) standards [10]. Metallic Ti peaks (JCPDS 44-1294) are identified for all electrodes due to penetration of X-rays through the oxide films, thus reaching the substrate [27,28]. Peaks linked to tetragonal RuO_2_ (JCPDS 40-1290) and tetragonal Sb_2_O_4_ (JCPDS 36-1163), as well as face-centered cubic metallic Pt (JCPDS 04–0802) are also identified on electrodes Ti/(RuO_2_)_66.5_-(Sb_2_O_4_)_28.5_-Pt_5_ and Ti/(RuO_2_)_63_-(Sb_2_O_4_)_27_-Pt_10_. In this context, a study has already corroborated highly crystalline Pt phases that form at temperatures above 200 °C [23]. The crystallite size (t) was calculated using the Scherrer equation (Equation (1)), where ʎ = 0.15418 nm is the X-ray wavelength, β is the total width at half height (FWHM) of the respective peaks, and θ is the position of the peak [29]. The peak at 2θ = 28°, presenting the highest intensity across all catalysts, was selected to determine the crystallite size [11]. The calculated sizes ranged from 17.3 nm to 41.6 nm, as indicated in Table 1. Notably, there was an observable increase in the crystallite size with a higher platinum concentration.

(1) $$t=\frac{0.90ʎ}{\beta \cos\theta }$$

**Figure 3:**
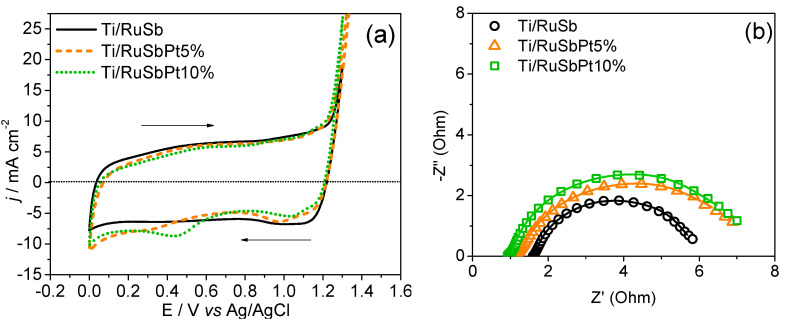
(**a**) Cyclic voltammograms and (**b**) Nyquist plots acquired using Ti/(RuO_2_)_70_-(Sb_2_O_4_)_30_, Ti/(RuO_2_)_66.5_-(Sb_2_O_4_)_28.5_-Pt_5_, and Ti/(RuO_2_)_63_-(Sb_2_O_4_)_27_-Pt_10_ electrodes. Electrolyte: H_2_SO_4_ 0.5 mol L^−1^. Scan rate: 50 mV s^−1^.

The electrochemical response of the developed electrodes is shown in Figure 3, which compares the cyclic voltammetry profiles and the electrochemical impedance spectra (EIS) of the three electrodes prepared in this study and studied in 0.5 mol L^−1^ H_2_SO_4_ electrolyte.

Cyclic voltammograms exhibit the typical profile of Ru-based electrodes, as illustrated in Figure 3a. In the case of electrodes containing Pt, the presence of reversal scan waves at 1.0 and 0.4 V vs. Ag/AgCl can be associated with platinum oxide reduction. The voltammetric charge (q*) is recorded within a potential range from 0.0 V to 1.3 V and can be used to estimate the electrochemically active surface area. The voltammetric charge values were consistently close, ranging between 281.6 and 298.1 mC cm^−2^. Compared to previously reported Ti/RuO_2_-Sb_2_O_4_ electrodes prepared via thermal decomposition in an electric furnace, the voltammetric charges in this study were significantly higher (97.11 mC cm^−2^) than those presented in this work [18].

In this study, the morphology factor (φ) was calculated based on the ratio between the internal (C_d,i_) and total (C_d_) surface areas, according to the methodology proposed by Da Silva et al. [30]. For all electrodes, the values of φ ≥ 0.84 (Table 2) (following the processing of results obtained in the voltammetric study shown in Figure S3) indicate electrodes with a large internal region accessible to the electrolyte.

Regarding EIS, our analysis reveals the presence of a well-defined semi-circle, consistent with observations from previous investigations of mixed metal oxide electrodes. As commonly reported in the literature [30], the most appropriate circuit model to describe the system is represented by R_Ω_(R_ct_C_dl_) [31]. In this circuit, ohmic resistance (R_Ω_), which is related mainly to the electrolyte solution and electrode contacts, is in series with the parallel combination of pseudo-capacitance (represented by a constant phase element–CPE) reflecting the electrical double layer (dl) behavior, and a charge transfer resistance (R_ct_) characterizing the interface between the MMO film and the electrolyte. CPEs are often employed for MMO electrodes to represent nonideal capacitance due to the inhomogeneity of the film surface, so-called Q_CPE(dl)_ [31]. The parameters obtained after fitting the EIS data are summarized in Table 3, demonstrating a good agreement between the proposed equivalent circuit and all impedance data, with a quality factor χ^2^ < 7 × 10^−4^. The Nyquist plot (Figure 3b) and R_ct_ values obtained by fitting the EIS data (Table 3) of the three electrodes indicate a trend of increased charge transfer resistance with increasing platinum content. Nonetheless, the implications of these enhanced properties on catalytic efficiency necessitate further validation in electrolyzer and fuel cell configurations.

Linear scanning voltammetry (LSV) experiments were conducted at a scan rate of 20 mV s^−1^ in a 0.5 M H_2_SO_4_ solution using three distinct electrodes: (a) Ti/(RuO_2_)_70_-(Sb_2_O_4_)_30_, (b) Ti/(RuO_2_)_66.5_-(Sb_2_O_4_)_28.5_-Pt_5_, and (c) Ti/(RuO_2_)_63_-(Sb_2_O_4_)_27_-Pt_10_. These experiments aimed to investigate the electrocatalytic behavior of the electrodes after they underwent 500 cycles. Figure S4 shows that all catalysts have a similar onset potential, approximately 1.2 V vs. Ag/AgCl, for oxygen evolution. This consistent performance across different electrode compositions indicates their high stability, demonstrating their potential for applications in reversible cells [32].

### 3.2. Electrolyzer Operation

**Figure 4:**
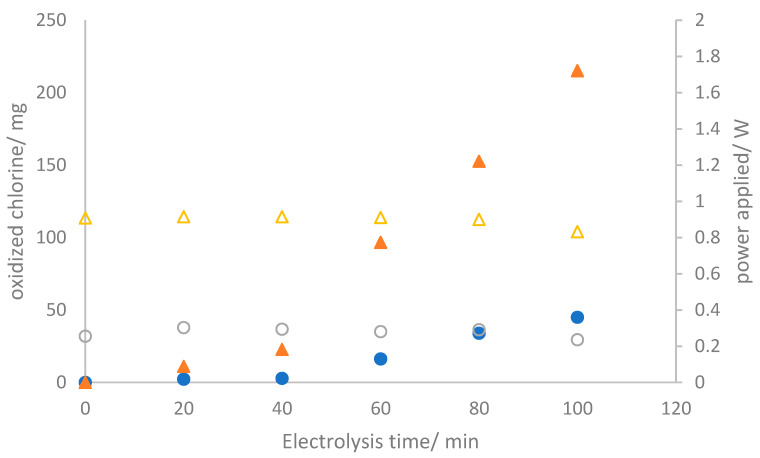
Temporal evolution of the oxidized chlorine species produced (● and ▲) and supplied power (○ and Δ) during the electrolysis of 2.0 mol L^−1^ sodium chloride aqueous solutions at (●) 50 mA cm^−2^ and (▲) 150 mA cm^−2^.

Following the physicochemical and electrochemical characterization of the three electrodes produced for this study, the evaluation of their performance under bulk conditions becomes imperative. In the context of the electrolyzer operation mode, two different galvanostatic conditions were fixed by maintaining operational current densities of 50 and 150 mA cm^−2^. Figure 4 illustrates the temporal evolution of the production of oxidized chlorine species (OCS) and the instantaneous energy powered during the two tests (which were carried out at current densities of 50 and 150 mA cm^−2^) in which a 2.0 mol L^−1^ sodium chloride solution is electrolyzed.

**Figure 5:**
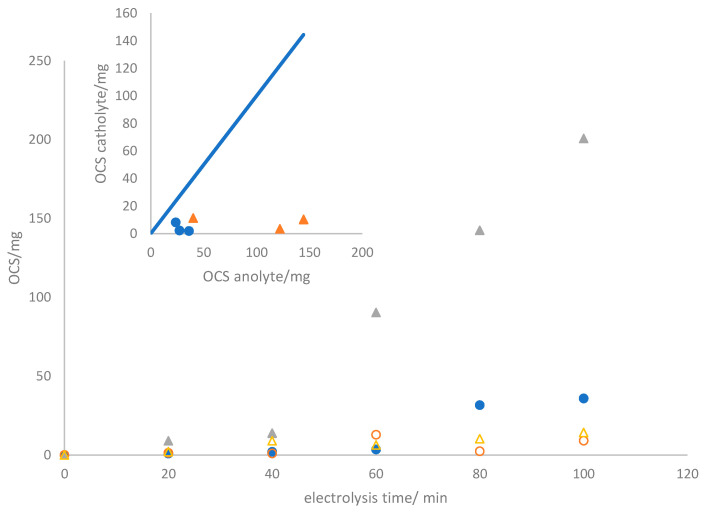
Time evolution of OCS in the anodic (● and ▲) and cathodic (○ and Δ) compartments during the electrolysis of 2.0 mol L^−1^ sodium chloride aqueous solutions at ● 50 mA cm^−2^, ▲150 mA cm^−2^. Inset: OCS values obtained from different tests performed with the same apparatus after 80 min of electrolysis.

As anticipated, the concentration of oxidized chlorine species continuously increases, reaching values of 44.96 and 214.95 mg after 100 min of electrolysis, corresponding to current charges passed of 0.083 and 0.25 Ah, respectively. The power consumption remains constant, indicating a stable cell voltage throughout the test. This suggests the absence of surface alterations or damage to the electrodes and membrane [33]. It is essential to note that oxidized chlorine species are detected not only in the anolyte but also in the catholyte. Significant amounts of OCS are detected in the catholyte, with values showing minimal dependence on the current density, a trend verified in Figure 5. The precise relationship between these values and anolyte concentrations is unclear, indicating a more intricate connection than a direct correlation.

This phenomenon can be elucidated in terms of the crossover of species from the anolyte to the catholyte, especially the OCS. The inset of Figure 4 presents the number of chlorine oxidant species that traverse the membrane after the same experimental duration in alternative tests performed using the same setup. The similarity of the amounts reaching the catholyte implies a crossover occurrence attributed to the membrane’s permeability to these species. Based on these data, the crossover rate is estimated to be 0.0245 ± 0.0167 mg OCS min^−1^ cm^−2^. In particular, the visual integrity of the membrane is retained throughout the process, and the cell voltage is kept almost constant, as previously indicated. These results indicate the absence of damage and a distinctive characteristic of the membrane. Addressing this crossover will be essential to enhance efficiency and merits further investigation.

**Figure 6:**
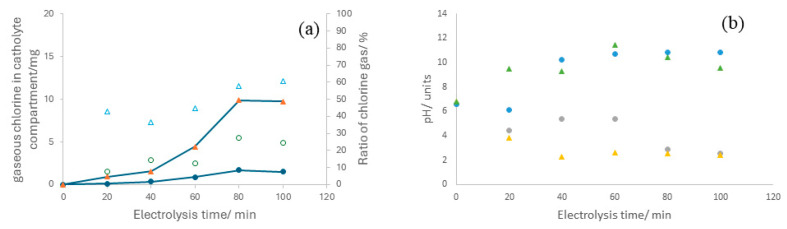
(**a**) Temporal evolution of gaseous OCS within the cathodic compartment during electrolysis of 2.0 mol L^−1^ sodium chloride aqueous solutions at current densities of (●) 50 mA cm^−2^ and (▲) 150 mA cm^−2^. Empty symbols represent the ratio of gaseous OCS, whereas full symbols denote the absolute amount of gaseous OCS. (**b**) time-course of the pH variations in the anolyte and catholyte during the electrolysis of 2.0 mol L^−1^ sodium chloride aqueous solutions at (●) 50 mA cm^−2^ and (▲) 150 mA cm^−2^.

Regarding the speciation of chlorine detected in the cathode, it is significant that a critical ratio is contained in the gas phase, which initially appears contradictory to the pH of the catholyte. Note that the hypochlorite anion is the primary expected chlorine oxidant species, not gaseous chlorine. Figure 6 illustrates the amounts of gaseous chlorine detected in the catholyte compartment gasometer along with the gaseous OCS to total OCS ratio, which approaches percentages of 30 and 60%, respectively, at current densities of 50 and 150 mA cm^−2^.

The only way to explain these vast ratios, considering the pH values in the anolyte and catholyte shown in Figure 6b, is by assuming that species crossover from anolyte to catholyte does not involve HClO but gaseous Cl_2_.

In previous studies on the same technology [23], hydrogen measurements were suggested in two approaches. The first method involved experimental hydrogen based on the gas volume collected in the associated gasometer within the catholyte compartment. The second approach utilized an indirect method hinging on the equilibrium of protons and hydroxyl ions. This approach considered significant reactions that affect pH, such as oxygen evolution at the anode (Equation (2)) and hydrogen evolution at the cathode (Equation (3)). It should be noted that the oxidation of chloride to chlorine (Equation (4)) does not inherently alter pH.

(2) $${2H}_{2}O\to O_{2}+4H^{+}+4e^{-}$$

(3) $${2H}_{2}O+{2e}^{-}\to H_{2}+{2OH}^{-}$$

(4) $$2{Cl}^{-}\to {Cl}_{2}+2e^{-}$$

**Figure 7:**
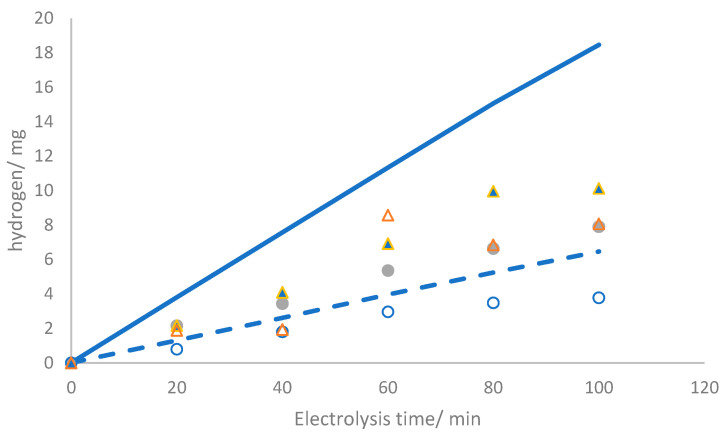
Production of hydrogen during electrolysis of 2.0 mol L^−1^ NaCl at (●, ○) 50 mA cm^−2^ and (▲, Δ) 150 mA cm^−2^. The dashed and continuous lines correspond to the theoretical expected values considering 100% faradaic efficiency at 50 and 150 mA cm^−2^, respectively. The empty symbols represent experimental points calculated from pH via the proton and hydroxyl ion balance, while the solid symbols denote experimental points derived from the cumulative gas content in the cathodic gasometer.

The hydrogen-produced hydrogen measured experimentally and calculated using the indirect method is shown in Figure 7. Noticeably, the values obtained are contradictory. In the test carried out at 50 mA cm^−2^, the quantification of hydrogen through the accumulation of gases surpasses the theoretical maximum efficiency illustrated by the dashed line, supporting the idea that the chlorine gas permeates the membrane. Conversely, the determination of hydrogen based on proton and hydroxyl balance yields significantly lower values than anticipated. This trend can be explained in terms of competition between the reduction of water and chlorine on the cathode surface, which is a more plausible mechanism.

Regarding the test results carried out at 150 mA cm^−2^, the convergence between the methods of hydrogen quantification is higher than at 50 mA cm^−2^ due to the appreciably reduced proportion of gaseous chlorine. It is noteworthy that the current density minimally influences the crossover of species and is primarily dependent on membrane characteristics.

In conclusion, these outcomes point out that, for chloralkaline technology, the use of membranes that prevent the crossover of chlorine is critical, given the pronounced impact of such crossover on the resultant outcome.

### 3.3. Fuel Cell Operation

**Figure 8:**
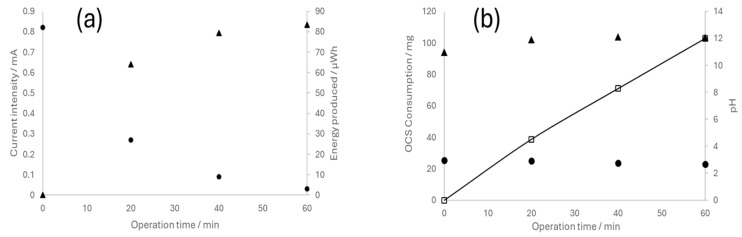
Operation of the reversible chloralkaline electrochemical cell as a fuel cell. (**a**) Current intensity (●) and energy produced (▲); (**b**) cathodic pH (▲), anodic pH (●), and OCS consumption (□).

Following the completion of the electrolytic experiments, the reversible operation of the electrochemical cell, namely, the fuel cell mode, was evaluated. For this investigation, the cells were connected to a multimeter to obtain voltage values and supplied with the hydrogen and chlorine previously accumulated in the compartments/gasometers during their performance as electrolyzers at a current density of 150 mA cm^−2^. Subsequently, changes in the different parameters over time were recorded. Data on the cell equipped with the catalyst examined in the previous section are presented in Figure 8.

Evidently, the system exhibits high performance, wherein the current decreases concomitantly with the consumption of the reagents. This observation confirms the feasibility of using reversible electrochemical cells to store energy in chloralkaline systems. The continuous depletion of OCS corroborates the sustained operation of the system, while fluctuations in the pH of the anodic and cathodic compartments (now occurring in the reverse way compared to electrolyzer operation) were also as expected.

### 3.4. Effect of Platinum Content on Reversible Cell Performance

Having established the feasibility of a reversible electrochemical cell based on the chloroalkaline process, we proceeded to assess the performance of the three electrodes prepared and characterized in this work. The tests conducted with the three distinct electrodes exhibited diverse responses, as discussed hereafter.

**Figure 9:**
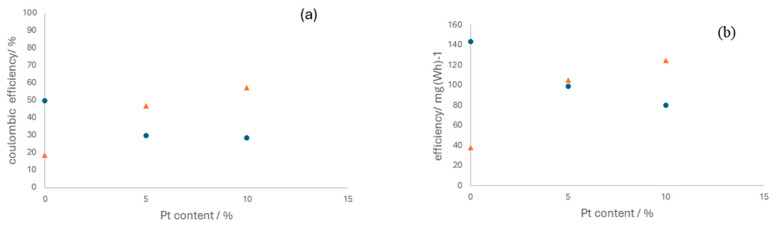
Influence of the platinum content in the electrocatalyst on the Coulombic efficiency (**a**) and energy efficiency (**b**). Operation current density: (●) 50 mA cm^−2^ and (▲)150 mA cm^−2^.

In terms of electrolyzer operation, the differences observed are associated with the effect of platinum on the selectivity of the anodic process only, specifically in promoting the oxidation of either chloride or water. Figure 9 shows the Coulombic and energy efficiencies attained in various tests, showing the same shape but with differences in values, which originated from the different energy consumption associated, in turn, with the resulting cell voltages.

The influence of platinum on the operation varies significantly depending on the current densities. At lower current densities, platinum appears to hinder chloride oxidation, leading to the evolution of oxygen as the predominant reaction. However, at higher current densities, the beneficial effect of platinum becomes apparent. This suggests that to optimize OCS production, it is advantageous to operate at higher current densities and use anodes with a higher platinum concentration. This observation is consistent with the insights derived from EIS, indicating that electrode capacities improve with increased platinum proportions.

**Figure 10:**
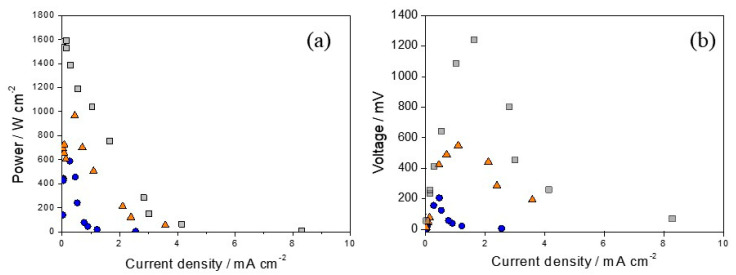
Polarization (**a**) and power curves (**b**) of fuel cells equipped with electrodes with different platinum ratios as cathodes: (●) Ti/(RuO_2_)_70_-(Sb_2_O_4_)_30_, (▲) Ti/(RuO_2_)_66.5_-(Sb_2_O_4_)_28.5_-Pt_5_, (■) Ti/(RuO_2_)_63_-(Sb_2_O_4_)_27_-Pt_10_.

In the context of fuel cell operation and the realm of fuel cell functionality, a comparative study was conducted, focusing on polarization and power curves, to assess the performance of electrocatalysts with varying concentrations of platinum serving as cathodes (Figure 10). The data revealed a notable enhancement in efficiency that was correlated with the increase in platinum content. This highlights the crucial role of careful control of platinum ratios in significantly improving the performance of reversible chloralkaline electrochemical cells.

We compared the performance of the electrodes that showed the highest efficiency in cell mode, as shown in Table 4, with the cell efficiency and maximum voltage values reported in other studies. Huskinson et al. [25] used a regenerative hydrogen chlorine fuel cell that incorporated an alloy (Ru_0.09_Co_0.91_)_3_O_4_ deposited on carbon as a chlorine electrode. The maximum power density obtained was 0.4 W cm^−2^, and the maximum voltage was 1.0 V, equivalent to 1000 mV, which is lower than the results of this study. In a more recent study, Carvela et al. [16] used a reversible electrochemical cell. They showed that the Ti/Ti_0.8_Pt_0.2_ electrode yielded a power density value of 3 mW cm^−2^ and a maximum voltage of 1300 mV, both similar to the values found in our study. In another study [24], the authors studied the efficiency of a reversible electrochemical cell using various electrodes such as Ti/Ru_0.5_Ir_0.5_O_2_, Ti/Ru_0.3_Ti_0.7_O_2_, and Ti/Ru_0.3_Ti_0.6_O_2_Pt_0.1_. Their investigation demonstrated that, during electrolysis, the Ti/Ru_0.3_Ti_0.6_O_2_Pt_0.1_ electrode exhibited superior performance compared to the other electrodes.

Conversely, in the fuel cell mode, the Ti/Ru_0.5_Ir_0.5_O_2_ electrode showed a maximum power efficiency of 6 mW cm^−2^, coupled with maximum current densities greater than 35 mA cm^2^. This behavior is intrinsically related to the composition of the electrode that directly affects the efficiency of the system. In another report [34], the impact of operational temperature on reversible cells was investigated, affirming its pivotal role in fuel cell performance. The research emphasized the significance of operating at ambient temperatures to preserve electrode efficiency. The electrode used for hydrogen electrochemistry was made of Pt/C (40% by weight) and was responsible for the hydrogen electrochemistry, whereas a commercial RuO_2_ catalyst was responsible for the chlorine reaction. In particular, at a temperature of 20 °C, the reversible cell yielded a current density of approximately 14 mA cm^−2^ at a potential of 0.5 V, simultaneously achieving a power density of around 70 mW cm^−2^. According to these analyses, the results of this study show a very high power efficiency and a maximum voltage in the fuel cell mode, highlighting the Ti/(RuO_2_)_63_-(Sb_2_O_4_)_27_-Pt_10_ electrode as a promising candidate for fuel cell applications.

The data were fitted using a least square procedure and the model outlined in Equation (5). This model is a useful simplification of Equation (6), where the terms E correspond to the Nernst equation (independent of the current density), *η* corresponds to the Butler–Volmer equation, which conveniently approximates the Tafel equation (Equation (7)) when the |*η*| > 0.1 V. In this model, parameter *b* is associated with kinetic information, and parameter *c* is associated with ohmic losses, while parameter *a* contains both kinetic and thermodynamic insights.

(5) V=a+b·log⁡j+c·j

(6) $$V=E_{a}+E_{c}+\eta_{a}+\eta_{c}+\sum_{k}I\cdot R_{k}$$

(7) $$j=j_{0}e^{b\cdot \eta }$$

The results of the fitting are shown in Table 5, revealing that the increase in the content of platinum favors the kinetics of the fuel cell, indicated by a decrease in parameter *b*. Moreover, the increase in platinum content also minimizes the ohmic losses, which is evident in the diminished value of the parameter *c*. Hence, according to the fitted parameters, the enhanced performance can be attributed to the synergistic effects of reduced electrical resistance as a result of increased platinum loading coupled with improved electrocatalytic efficiency, as previously stated from the EIS analyses carried out on the different electrodes.

The findings emphasize that electrodes manufactured from MMO are fundamental for investigating their performance in reversible electrochemical cells. Likewise, electrodes containing Pt at high current densities can promote high performance in terms of reversible chloralkaline electrochemical cells.

## 4. Conclusions

This study investigated the operational efficiency and stability of a chlor-alkali reversible electrochemical cell employing three distinct ternary MMO electrodes containing ruthenium, antimony, and low amounts of platinum. The utilization of a synthesis methodology that combined IL and microwave irradiation to produce the MMO electrodes led to electrodes with a distinctive “cracked mud” appearance, characteristic of thermally decomposed oxide layers, and the successful incorporation of Ru, Sb, and Pt elements. Notably, semiquantitative identification of deposited metals on the electrodes revealed molar ratios that closely match those of the precursor solutions. Based on data from the study, it was demonstrated that the reversible electrochemical cell could function effectively in both electrolyzer and fuel cell modes, operating at distinct current densities of 50 and 150 mA cm^−2^. Notably, the concentration of oxidized chlorine species increased with elevated current density, from 44.96 to 214.95 mg, after 100 min of electrolysis.

Furthermore, evidence indicated the occurrence of the cross-migration of oxidizing species between both compartments, with an estimated crossing rate of 0.0245 ± 0.0167 mg OCS min^−1^ cm^−2^. This cross-migration of species from the anolyte to the catholyte was associated with gaseous Cl_2_. In fuel cell mode, the system showed remarkable efficiency in electricity generation. Examination of the electrode behavior highlighted the positive impact of platinum, particularly in the production of OCS under high current densities. On the other hand, the increase in platinum content exhibited a favorable influence on the kinetics and performance of reversible chloralkaline electrochemical cells. Hence, this study advances our comprehension of reversible electrochemical cells and presents possibilities for further exploration and refinement. Therefore, this report demonstrated that the interplay of innovative synthesis strategies of electrodes and electrochemical engineering could result in promising and sustainable technologies for energy conversion.

## Figures and Tables

**Table 1:** Nominal and experimentally determined semiquantitative concentrations of the electrodes studied, assessed through EDS and ICP-AES techniques, and parameters derived from XRD data of width at half height and crystallite size determined using the Scherrer equation.

Electrode	Nominal Composition (mol%)			Experimental Composition from EDS (mol%)			Experimental Composition from ICP (mol%)			XRD	
	Ru	Sb	Pt	Ru	Sb	Pt	Ru	Sb	Pt	FWHM (rad)	Crystallite Size (nm)
Ti/(RuO_2_)_70_-(Sb_2_O_4_)_30_	70	30	-	75.5	24.5	-	69.4	30.6	-	0.2362	17.3
Ti/(RuO_2_)_66.5_-(Sb_2_O_4_)_28.5_-Pt_5_	66.5	28.5	5	67.0	27.5	5.5	84.9	12.5	2.6	0.3936	20.8
Ti/(RuO_2_)_63_-(Sb_2_O_4_)_27_-Pt_10_	63	27	10	66.2	23.6	10.2	60.8	22.7	16.5	0.1968	41.6

**Table 2:** Dependence of total (C_d_), external (C_d,e_), and internal (C_d,i_) differential capacitance and morphology factor, φ.

Electrode	q*(mC cm^−2^)	C_d_ (mF cm^−2^)	C_d,e_ (mF cm^−2^)	C_d,i_ (mF cm^−2^)	φ (C_d,i_/C_d_)
Ti/(RuO_2_)_70_-(Sb_2_O_4_)_30_	298.1	46.1	17.3	39.1	0.84
Ti/(RuO_2_)_66.5_-(Sb_2_O_4_)_28.5_-Pt_5_	290.4	52.9	20.8	46.44	0.88
Ti/(RuO_2_)_63_-(Sb_2_O_4_)_27_-Pt_10_	281.6	47.03	41.6	42.03	0.89

**Table 3:** Summary of fitted EIS data for different mixed metal oxide anodes containing different platinum contents recorded at 1.22 V vs. Ag/AgCl.

Electrode	R_Ω_/Ω	Q_CPE(dl)_/F	R_tc_/Ω	η	χ^2^
Ti/(RuO_2_)_70_-(Sb_2_O_4_)_30_	1.65	0.28	4.3	0.90	0.0006
Ti/(RuO_2_)_66.5_-(Sb_2_O_4_)_28.5_-Pt_5_	1.25	0.19	6.1	0.84	0.0006
Ti/(RuO_2_)_63_-(Sb_2_O_4_)_27_-Pt_10_	1.03	0.18	6.3	0.84	0.0007

**Table 4:** Comparison of the recent research regarding fuel cell operation. Performance is described in terms of power density and maximum cell voltage.

Electrode	Power Density (W cm^−2^)	Maximum Cell Voltage (mV)	Reference
Ti/(RuO_2_)_63_-(Sb_2_O_4_)_27_-Pt_10_	1.6	1300	This work
(Ru_0.09_Co_0.91_)_3_O_4_	0.4	1000	[25]
Ti/Ru_0.5_Ir_0.5_O_2_	0.006	−	[24]
Pt/C	0.007	−	[34]

**Table 5:** Fuel cell kinetic parameters at different mixed metal oxide anodes containing different platinum contents. Parameter *a* indicates kinetic and thermodynamic insight, and Tafel slopes, *b*, and ohmic losses are represented by *c*.

	Platinum Content (%)		
Parameter	0	5	10
*a*/V	2053.22	1411.94	1454.15
*b*/V dec^−1^	1141.01	483.07	131.13
*c*/Ω	−2801.24	−641.32	−404.87

## Data Availability

Data are contained within the article and Supplementary Materials.

## Supplementary Materials

The following supporting information can be downloaded at: https://www.mdpi.com/article/10.3390/nano14080693/s1, Figure S1: Schematic representation of the experimental setup used in this study, featuring gasometers in the anode and cathode compartments, power supply, electrochemical reversible cell, and a multichannel peristaltic pump. The right image provides an enlarged view of the reactor; Figure S2: XRD diffraction patterns of the Ti/(RuO_2_)_70_-(Sb_2_O_4_)_30_, Ti/(RuO_2_)_66.5_-(Sb_2_O_4_)_28.5_-Pt_5_, and Ti/(RuO_2_)_63_-(Sb_2_O_4_)_27_-Pt_10_ electrodes; Figure S3: Cyclic voltammograms recorded in a 0.5 M H_2_SO_4_ solution during repetitive potential cycles at a scan rate ranging from 10 to 300 mV s^−1^ (a,c,e). The correlation between the voltammetric capacitive responses is shown in (b,d,f) for the electrodes (a,b) Ti/(RuO_2_)_70_-(Sb_2_O_4_)_30_, (c,d) Ti/(RuO_2_)_66.5_-(Sb_2_O_4_)_28.5_-Pt_5_, and (e,f) Ti/(RuO_2_)_63_-(Sb_2_O_4_)_27_-Pt_10_; Figure S4: Linear sweep voltammetry profiles recorded at a scan rate of 20 mV s^−1^ in a 0.5 M H_2_SO_4_ solution before cycling and after 500 cycles at different electrodes studied in this work.
