# Supplementary material for: Improved Operation of Chloralkaline Reversible Cells with Mixed Metal Oxide Electrodes Made Using Microwaves
Source: Nanomaterials (Basel). 2024 Apr 17;14(8):693. doi: 10.3390/nano14080693 (PMC11054361; doi:10.3390/nano14080693)
Supplement: Supplementary file 1 [file nanomaterials-14-00693-s001.zip › nanomaterials-2824517-supplementary.pdf]

Supplementary Materials

Improved Operation of Chloralkaline Reversible Cells with Mixed Metal Oxide Electrodes Made Using Microwaves

Jamylle Y. C. Ribeiro [1,2,3], Gessica O. S. Santos [3,4], Aline R. Dória [1,4], Iñaki Requena [3], Marcos R. V. Lanza [4], Giancarlo R. Salazar-Banda [1,2], Katlin I. B. Eguiluz [1,2], Justo Lobato [3,*] and Manuel A. Rodrigo [3,*]

1 Electrochemistry and Nanotechnology Laboratory, Institute of Technology and Research (ITP), Aracaju 49032-490, SE, Brazil; jamyllerib@gmail.com (J.Y.C.R.); alinerdoria@gmail.com (A.R.D.); gianrsb@gmail.com (G.R.S.-B.); katlinbarrios@gmail.com (K.I.B.E.)

2 Graduate Program in Processes Engineering (PEP), Tiradentes University, Aracaju 49032-490, SE, Brazil

3 Chemical Engineering Department, Faculty of Chemical Sciences and Technologies, Universidad Castilla-La Mancha, 13004 Ciudad Real, Spain; gessicasantiago@usp.br (G.O.S.S.); inaki.requena@uclm.es (I.R.)

4 São Carlos Institute of Chemistry, University of São Paulo, São Carlos 13566-590, SP, Brazil; marcoslanza@usp.br

* Correspondence: justo.lobato@uclm.es (J.L.); manuel.rodrigo@uclm.es (M.A.R.)

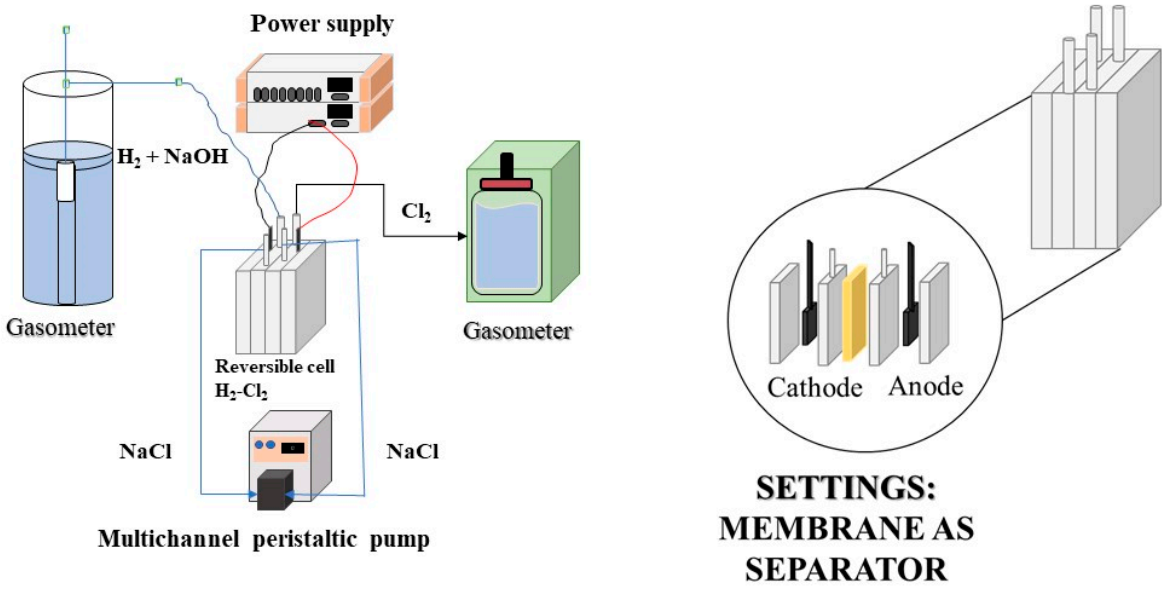

Figure S1. Schematic representation of the experimental setup used in this study, featuring gasometers in the anode and cathode compartments, power supply, electrochemical reversible cell, and a multichannel peristaltic pump. The right image provides an enlarged view of the reactor.

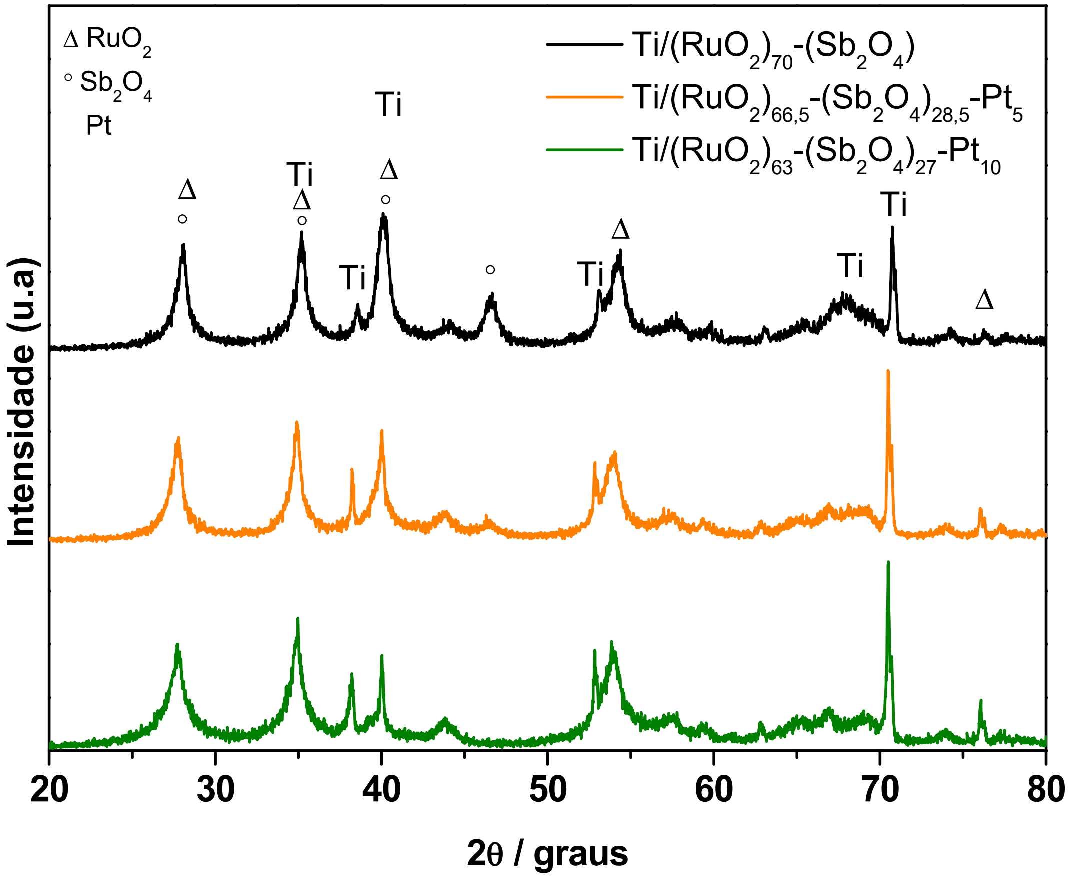

Figure S2. XRD diffraction patterns of the Ti/$(RuO_2)_{70}$-$(Sb_2O_4)_{30}$, Ti/$(RuO_2)_{66.5}$-$(Sb_2O_4)_{28.5}$-Pt_5, and Ti/$(RuO_2)_{63}$-$(Sb_2O_4)_{27}$-Pt_{10} electrodes.

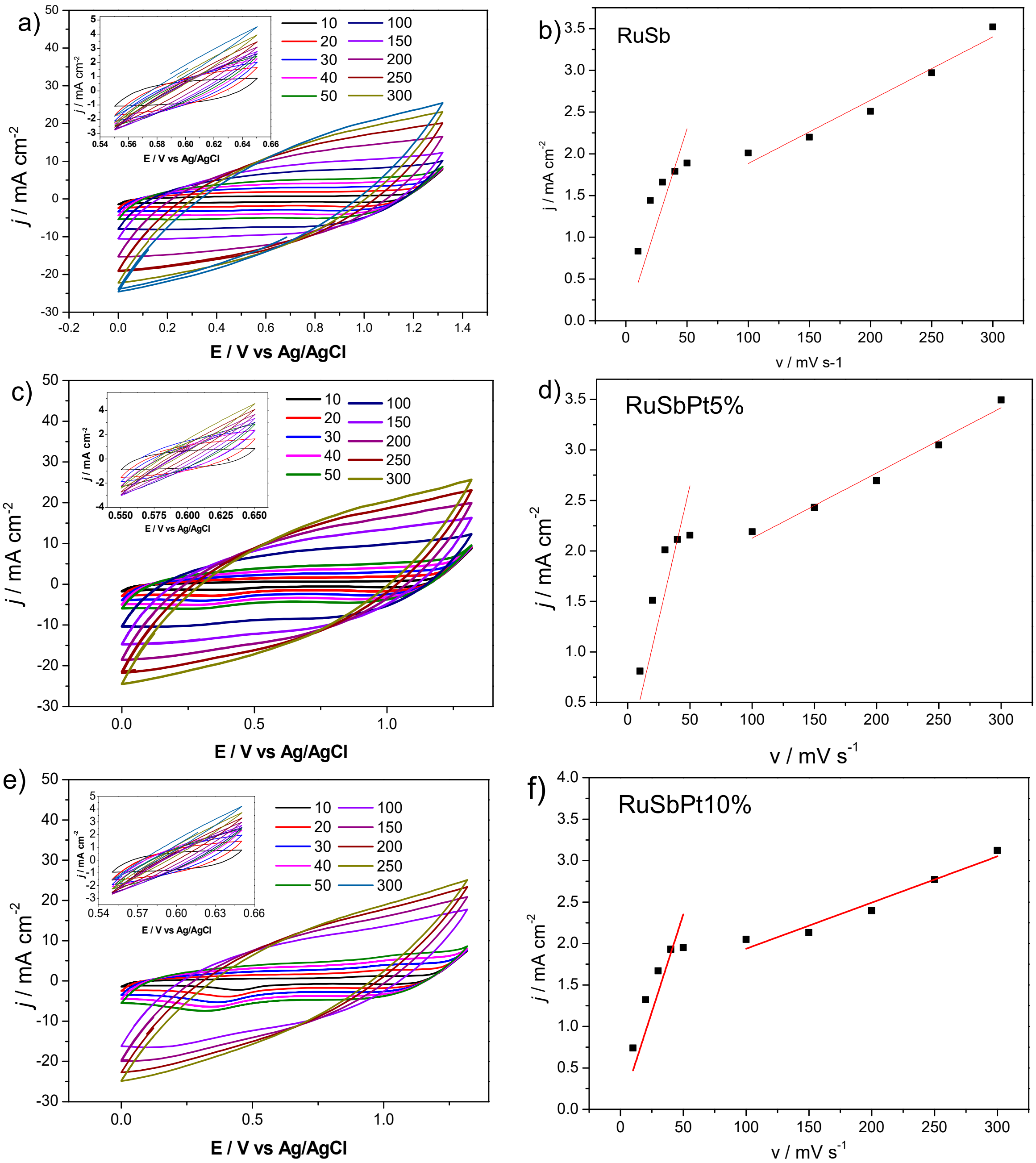

Figure S3. Cyclic voltammograms recorded in a 0.5 M H_2SO_4 solution during repetitive potential cycles at a scan rate ranging from 10 to 300 mV s^{-1} (**a**,**c**,**e**). The correlation between the voltammetric capacitive responses is shown in (**b**,**d**,**f**) for the electrodes (**a**,**b**) Ti/$(RuO_2)_{70}$-$(Sb_2O_4)_{30}$, (**c**,**d**) Ti/$(RuO_2)_{66.5}$-$(Sb_2O_4)_{28.5}$-Pt_5, and (**e**,**f**) Ti/$(RuO_2)_{63}$-$(Sb_2O_4)_{27}$-Pt_{10}.

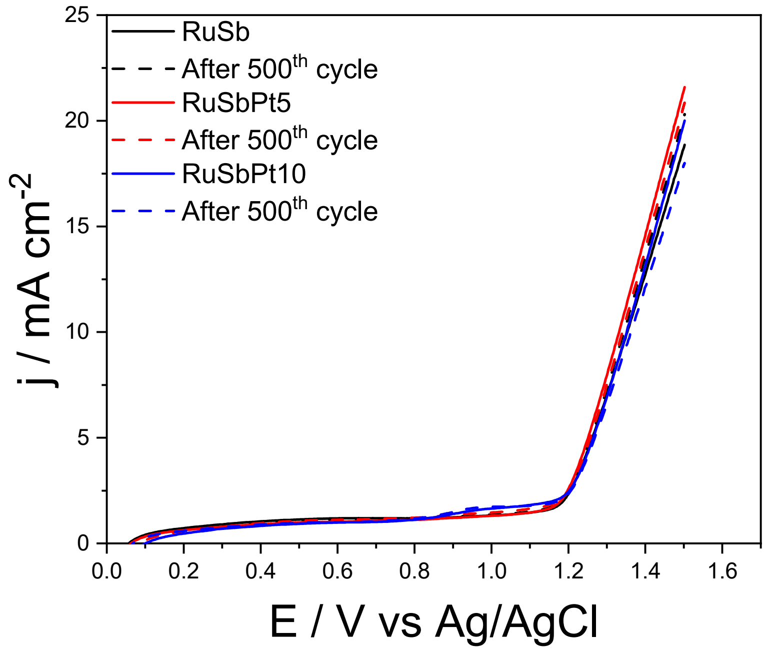

Figure S4. Linear sweep voltammetry profiles recorded at a scan rate of 20 mV s^{-1} in a 0.5 M H_2SO_4 solution before cycling and after 500 cycles at different electrodes studied in this work.
